# Inverse Correlation Between Grip Strength and Serum Phosphorus: A Retrospective Observational Study in Japanese Elderly with Poorly Controlled Type 2 Diabetes

**DOI:** 10.3390/geriatrics5020033

**Published:** 2020-05-19

**Authors:** Sho Tanaka, Masahiro Takubo, Genta Kohno, Masaru Kushimoto, Jin Ikeda, Katsuhiko Ogawa, Yutaka Suzuki, Masanori Abe, Hisamitsu Ishihara, Midori Fujishiro

**Affiliations:** 1 Division of Nephrology, Hypertension and Endocrinology, Department of Internal Medicine, Nihon University School of Medicine, Tokyo 173-8610, Japan; tanaka.sho@nihon-u.ac.jp (S.T.); abe.masanori@nihon-u.ac.jp (M.A.); 2 Department of Internal Medicine, Nihon University Hospital, Tokyo 101-8309, Japan; takubo.masahiro@nihon-u.ac.jp (M.T.); kushimoto.masaru@nihon-u.ac.jp (M.K.); septembre.99@gmail.com (J.I.); ogawa.katsuhiko@nihon-u.ac.jp (K.O.); suzuki.yutaka@nihon-u.ac.jp (Y.S.); 3 Division of Diabetes and Metabolic Diseases, Department of Internal Medicine, Nihon University School of Medicine, Tokyo 173-8610, Japan; kohno.genta@nihon-u.ac.jp (G.K.); ishihara.hisamitsu@nihon-u.ac.jp (H.I.); 4 Division of Neurology, Department of Medicine, Nihon University School of Medicine, Tokyo 173-8610, Japan

**Keywords:** diabetes mellitus, geriatrics, hand strength, phosphorus, sarcopenia

## Abstract

The aim of this study was to investigate factors associated with sarcopenia among elderly patients with poorly controlled diabetes mellitus (DM). We retrospectively analyzed 41 patients with type 2 DM, aged ≥65 years who required diabetes education hospitalization. Patients were classified into two groups according to the presence or absence of a weakened hand grip, and clinical characteristics were compared. Patients with a weakened hand grip (*n* = 21) scored worse on a mini-mental state examination (24.3 vs. 26.5, *p* = 0.04), showed a higher prevalence of diabetic peripheral neuropathy (76% vs. 40%, *p* = 0.03), and had a higher serum phosphorus concentration (3.8 vs. 3.3 mg/dL, *p* < 0.01) compared to those without a weakened hand grip (*n* = 20). The serum phosphorus concentration was inversely correlated to hand grip strength (*r* = −0.501, *p* < 0.001) among the total of 41 patients. This inverse association was also confirmed after adjusting the effects of estimated glomerular filtration rate, age, and glycated hemoglobin. Thus, cognitive impairment, diabetic peripheral neuropathy, and high serum phosphorus concentrations are associated with hand grip weakness in elderly patients with type 2 DM.

## 1. Introduction

Sarcopenia is an aging-associated muscle disease comprised of the following factors: low muscle strength, low muscle quantity or quality, and low physical performance [1]. In clinical practice, the measurement of grip strength is recommended for the assessment of sarcopenia since this is highly predictive [1]. Frailty is a condition that leads to increased vulnerability to dependency and mortality with multifactorial etiologies. It is clinically characterized by weight loss, exhaustion, low physical activity, slow gait speed, and muscle weakness that is mainly evaluated by grip strength [2]. Sarcopenia and frailty often overlap and co-exist because muscle weakness and poor physical performance are common in these two concepts and are associated with diverse adverse outcomes [1,2].

An increasing proportion of the elderly population have diabetes mellitus, which is a major problem in public health since complications due with geriatric syndromes often arise in such patients, who consequently require personalized medical care. Patients with diabetes mellitus are prone to frailty and sarcopenia, with this disease being recognized as an established risk for physical disability and disability in activities of daily living [3,4]. Decreased insulin activity, malnutrition, low-grade inflammation, decreased insulin-like growth factor-1, a low testosterone level, and vitamin D deficiency are thought to be involved in the development of frailty and sarcopenia [5]. In clinical practice, poor glycemic control predicts a decline in muscle performance in diabetic patients [6,7]. Nevertheless, the pathophysiology of the association between insufficient glycemic control and developmental mechanisms of frailty and sarcopenia has not been fully elucidated. This study aimed to investigate the clinical factors associated with sarcopenia in patients with poorly controlled type 2 diabetes mellitus.

## 2. Materials and Methods

### 2.1. Selection of Participants

This was a retrospective cross-sectional study. We investigated patients with poorly controlled diabetes mellitus who required education by means of a hospital program to reinforce glucose-lowering treatments in order to achieve adequate glycemic control. A total of 43 patients with type 2 diabetes mellitus, aged 65 years and over, who were admitted to Nihon University Hospital for diabetes education between August 2018 and July 2019, were potentially eligible for this study. We excluded two patients with missing data on the measurement of grip strength, and thus 41 patients in total were enrolled.

Clinical data were extracted by chart review. We classified patients into two groups by dominant hand grip strength according to the criteria of the Asian Working Group for Sarcopenia [8]. Patients with a hand grip strength <26 kg in men and <18 kg in women were classified into a lower hand grip strength (LHGS) group, while the remaining patients were included in a higher hand grip strength (HHGS) group.

### 2.2. Patients’ Characteristics

We studied patients’ characteristics comprising dominant hand grip strength, mini-mental state examination (MMSE) score, age, sex, body mass index (BMI), waist circumference, and blood pressure. The duration of diabetes mellitus, a family history of diabetes mellitus, current smoking, habitual alcohol use, uses of insulin and statins, a history of hypertension and dyslipidemia, and a history of cardiovascular, cerebrovascular, and peripheral artery diseases were also investigated.

### 2.3. Clinical Parameters: Diabetic Microangiopathy and Atherosclerosis

We then studied diabetic microangiopathies and surrogate markers for atherosclerosis. Diabetic peripheral neuropathy (DPN) was diagnosed using diagnostic criteria proposed by a diabetic neuropathy study group in Japan [9]. The coefficient of variation of the R-R interval (CVR-R) and prevalence of CVR-R of less than 2% were assessed for diabetic autonomic neuropathy. The existence of diabetic retinopathy was assessed by ophthalmologists based on Davis staging. Staging of diabetic nephropathy was according to the classification of diabetic nephropathy 2014 [10]. The estimated glomerular filtration rate (eGFR) for Japanese was calculated as follows:$\;194\times {\mathrm{Creatinine}}^{-1.094}\times {\mathrm{Age}}^{-0.287}\left(\times 0.739\;\mathrm{if}\;\mathrm{female}\right)$. Maximum intima-media thickness (IMT) and the average of left and right cardio-ankle vascular indexes (CAVI) were also investigated. Furthermore, minimal and average values of left and right ankle-brachial indexes (ABI) were studied.

### 2.4. Laboratory Parameters

The laboratory parameters examined were as follows: glycated hemoglobin (HbA1c) and glycated albumin (GA) for assessment of glycemic control; total lymphocyte count, hemoglobin, and albumin for nutrition markers; aspartate aminotransferase and alanine aminotransferase for liver function; serum creatinine and eGFR for kidney function; sodium, potassium, chloride, corrected calcium, and phosphorus for electrolytes; total cholesterol, high-density lipoprotein cholesterol, low-density lipoprotein cholesterol, and triglyceride for a lipid panel; and uric acid. Levels of low-density lipoprotein cholesterol were assessed by direct measurement and the Friedewald formula: Total cholesterol-High-density lipoprotein cholesterol-Triglyceride/5.

### 2.5. Statistical Analysis

Data are presented as mean ± standard deviation, or as a number or percentage. To determine the significance of differences of continuous variables between the two groups, we used Student’s *t*-test, a Welch *t*-test, or a Mann-Whitney U-test as appropriate. We use Fisher’s exact test for comparing numbers or percentages. Pearson’s correlation coefficient was used to determine the associations of serum phosphorus concentration and MMSE with hand grip strength. We use multiple linear regression analysis to evaluate the association between grip strength and serum phosphorus concentration with adjusting the effects of eGFR, age, BMI, and HbA1c.

All statistical analyses were performed using EZR (Saitama Medical Center, Jichi Medical University, Saitama, Japan), which is a graphical user interface for R (The R Foundation for Statistical Computing, Vienna, Austria) [11]. Throughout, *p* < 0.05 was considered statistically significant. The appropriate sample size is not known because this is an exploratory study.

### 2.6. Ethical Considerations

This study complied with the Declaration of Helsinki and was approved by the Institutional Review Board of Nihon University Hospital. A requirement for written informed consent was waived because this study was a retrospective observational one.

## 3. Results

The background characteristics of all patients studied are shown in Table 1. LHGS and HHGS groups included 21 and 20 participants, respectively. The mean age, and male to female sex ratio did not differ between the two groups. Hand grip strength among males, females, and total participants was significantly lower in the LHGS group than in the HHGS group (*p* < 0.001 for all). LHGS group patients also showed significantly lower MMSE scores than those in the HHGS group (24.3 ± 3.7 vs. 26.5 ± 3.2, *p* = 0.04), although data was lacking for several patients. Correlations between MMSE scores and hand grip strength did not achieve statistical significance (Pearson’s correlation coefficient of *r* = −0.226, *p* = 0.21, Supplementary Figure S1). Although not statistically significant, patients in the LHGS group tended to have a smaller waist circumference (90.4 ± 8.9 cm vs. 95.1 ± 12.5 cm, *p* = 0.18) and lower BMI (24.2 ± 3.5 kg/m^2^ vs. 25.5 ± 4.1 kg/m^2^, *p* = 0.30) than those in HHGS group. No marked difference was noted in other parameters between the two groups. LHGS group patients showed poorer cognitive function compared to those in the HHGS group.

The co-existence of diabetic microangiopathy and surrogate markers of atherosclerosis are shown in Table 2. Only a few patients showed stage 4 nephropathy, where the eGFR was <30 mL/min/1.73 m^2^; patients in this cohort were not classified as stage 5, which was ongoing dialysis therapy. The prevalence of DPN was significantly higher in the LHGS group (76% vs. 40%, Odds Ratio: 4.80, 95% confidence interval: 1.25, 18.40, *p* = 0.03). Prevalence rates for patients with CVR-R < 2.0% (56% vs. 27%, *p* = 0.18) and diabetic retinopathy (50% vs. 30%, *p* = 0.27) tended to be higher, and patients with pre-nephropathy (19% vs. 40%, *p* = 0.18) tended to be lower in the LHGS group, although these did not reach statistical significance. The levels of surrogate markers of atherosclerosis that included IMT, ABI, and CAVI also did not differ between the two groups. Thus, patients in the LHGS group had a greater rate of DPN than those in the HHGS group.

**Figure 1:**
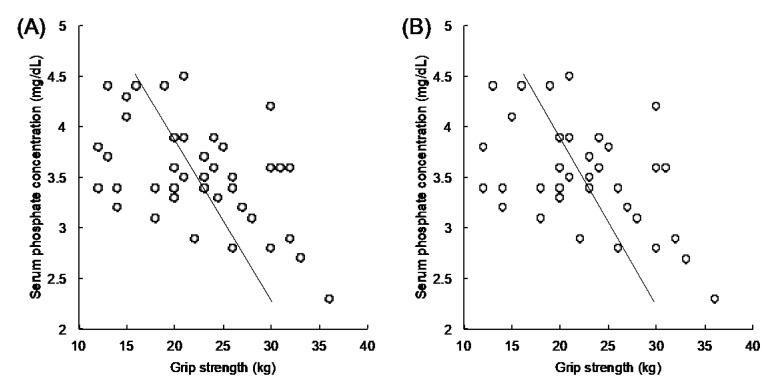
Correlation analysis between serum phosphorus concentration and hand grip strength. The vertical axis indicates the serum phosphorus concentration (mg/dL). The horizontal axis indicates grip strength (kg). Any correlations seen were validated using Pearson’s correlation coefficient. An inverse correlation is observed between the serum phosphorus concentration and hand grip strength in (**A**) total patients (*r* = −0.501, 95% confidence interval: −0.700, −0.228, *p* < 0.001), and (**B**) patients with eGFR ≥ 30 mL/min/1.73 m^2^; higher hand grip strength, *n* = 18 and lower hand grip strength, *n* = 18 (*r* = −0.501, 95% confidence interval: −0.712, −0.207, *p* = 0.002). eGFR, estimated glomerular filtration rate.

Table 3 shows the characteristics of laboratory parameters. Although glycemic control was not good in each group, differences in levels of HbA1c (8.8 ± 1.5% vs. 8.9 ± 1.4%, *p* = 0.87) and GA (25.6 ± 5.7% vs. 26.3 ± 6.3%, *p* = 0.73) were not noted between LHGS and HHGS groups. Nutritional laboratory markers, liver function, uric acid, and lipid panel and kidney function parameters did not differ between the two groups. Regarding electrolytes, the LHGS group showed a significantly higher serum phosphorus concentration than the HHGS group (3.8 ± 0.4 mg/dL vs. 3.3 ± 0.5 mg/dL, *p* < 0.01). Figure 1 illustrates the relationship between the serum phosphorus concentration and grip strength. The serum phosphorus concentration inversely correlated with grip strength (*r* = −0.501, *p* < 0.001) among all 41 patients in this study (Figure 1A). This inverse correlation was also confirmed (*r* = −0.501, *p* = 0.002) even after excluding patients with eGFR < 30 mL/min/1.73 m^2^ (Figure 1B). Additionally, multiple linear regression analysis demonstrated significant negative association between grip strength with serum phosphorus concentration after adjusting the effects of eGFR, age, and HbA1c (Table 4). Hence, patients with LHGS showed higher serum phosphorous concentrations than those in the HHGS group.

## 4. Discussion

The association between poor glycemic control, and frailty and sarcopenia has not been fully elucidated. This study investigated the clinical characteristics of Japanese elderly patients with type 2 diabetes mellitus and muscle weakness as assessed by dominant hand grip strength.

We found that weakened hand grip strength was associated with a decline in cognitive ability, the co-existence of DPN, and a high serum phosphorus concentration. Additionally, the serum phosphorus concentration inversely correlated with hand grip strength in this cohort of elderly patients with type 2 diabetes mellitus.

Worse cognitive function, as assessed by MMSE, in the LHGS group compared with the HHGS group is consistent with previous reports involving community-dwelling elderly. A recent meta-analysis showed that poor physical functions, including weakened grip strength, were associated with cognitive impairment [12]. Furthermore, a longitudinal study showed that weakened hand grip strength predicts cognitive decline among the Japanese population [13]. Our study indicates an association between physical and cognitive impairment also exists in the diabetic elderly.

In this study, the LHGS group had a relatively low BMI and small waist circumference, despite similar glycemic control, in comparison with the HHGS group. The association between somatotype and physical disability in the community-dwelling elderly has been repeatedly investigated, but results have been inconsistent; low or high BMI, or both, were reported to be risk factors for physical impairments [14,15,16]. Recently, the clinical characteristics of sarcopenic patients with diabetes mellitus were investigated in outpatient care; patients with sarcopenia showed a significantly lower BMI than those without sarcopenia, with the prevalence rate of sarcopenia increasing with decreasing BMI [17]. Because this previous study was conducted in an outpatient care setting, glycemic control was better (HbA1c 7.04 ± 1.02% for total, 7.04 ± 1.03% for non-sarcopenic and 7.02 ± 1.00% for sarcopenic patients) than in our present study, which investigated patients requiring diabetic education hospitalization [17]. Thus, regardless of glycemic control, a low BMI may be a risk factor for sarcopenia in diabetic patients.

In this study, DPN was more prevalent in the LHGS than HHGS group. The co-existence of diabetic neuropathy was found to be associated with weakened muscle strength and a decreased muscle mass volume of the lower extremities in type 2 diabetes mellitus [18,19]. In contrast, the association between diabetes mellitus and hand dysfunction was insufficiently validated. While a previous meta-analysis showed hand grip strength, pinch strengths, and dexterity tended to be worse in patients with type 2 diabetes than in healthy people, differences were not statistically significant [20]. Hand grip strength were found not to differ between type 2 diabetes patients, with and without DPN, in a Brazilian population [21]. In contrast to this previous report, our study showed an association between weakened hand grip strength and DPN in a Japanese population. Ethnicity-related differences may be one possible explanation for this discrepancy, but further study is required to comprehend the developmental mechanism behind weakened grip strength.

A previous study has reported that even within the normal range, elevated serum phosphorus levels were associated with increasing risks of acute kidney injury, end stage renal disease, and all-cause mortality [22]. Although average phosphorus values were within normal range in both groups of subjects assessed in the present study, the significantly higher serum phosphorus concentration in the LHGS compared to HHGS group and inverse correlation between serum phosphorus level and grip strength are striking discoveries. These indicate that high serum phosphorus is associated with sarcopenia in elderly patients with diabetes mellitus. Although the role of phosphorus in muscle contraction has been assessed in vitro, scant research exists on the association between the serum phosphorus level and muscle strength in humans [23,24]. Indeed, this issue was first described, to the best of our knowledge, by Chen et al. in 2018 [25]. As with the observations of our study, an inverse association between serum phosphorus and muscle strength was also noted, with a high phosphorus level predicting muscle weakness among participants investigated in a National Health and Nutrition Examination Survey (NHANES) involving an American population [25]. Our study suggests that this inverse association also relates to the Japanese population.

One possible explanation for a higher serum phosphorus concentration being linked to weakened muscle strength may be insufficient levels of fibroblast growth factor 23 (FGF23) and Klotho, its cofactor. The serum phosphorus concentration is determined by dietary intake and renal excretion in the steady-state. FGF23, Klotho, vitamin D, and parathyroid hormone are humoral factors that regulate phosphorus homeostasis [26]. FGF23 and Klotho mainly maintain urinary phosphorus excretion; any depleted activity of these molecules leads not only to hyperphosphatemia, but also to premature aging [27]. A higher FGF23 level is independently related to the prevalence of frailty in the community-dwelling elderly, even after adjustment for bone mineral metabolic markers such as phosphorus, calcium, parathyroid hormone, and vitamin D. Thus, endo-organ resistance to FGF23 in patients with frailty has been suggested [28].

In contrast, the involvement of vitamin D in an inverse correlation between phosphorus and muscle weakness has been uncertain, as an inverse association still existed after adjustment for the serum vitamin D concentration in a NHANES cohort; the vitamin D concentration did not differ between Japanese diabetic elderly with and without sarcopenia [17,25]. Although a high parathyroid hormone level may be a risk factor for sarcopenia, the serum phosphorus concentration in such a setting remains unknown [29]. Taken together, serum phosphorus is an attractive candidate as a conveniently measurable surrogate biomarker for sarcopenia, and the underlying pathophysiology should be investigated in future studies.

This study had several limitations. First, we investigated patients with poorly controlled diabetes; thus, the generalizability of an inverse correlation between serum phosphorus concentration and grip strength should be validated, in any future study, in patients without diabetes or those with adequately controlled diabetes mellitus. Second, a causal relationship between the serum phosphorus concentration and grip strength could not be established because this was a retrospective observational study. Third, since this is an exploratory study and not conducted to specifically investigate the association between phosphorus regulation and sarcopenia, we did not study factors influencing phosphorus regulation, such as FGF23, klotho, vitamin D, parathyroid hormone, and dietary intakes. Therefore, future prospective studies will be required in order to clarify interactions between these factors and sarcopenia in a larger population.

## 5. Conclusions

In conclusion, this study revealed that cognitive impairment, DPN, and a high serum phosphorus concentration are associated with weakened grip strength in Japanese elderly with type 2 diabetes mellitus. The serum phosphorus concentration was found to be inversely correlated with grip strength in this patient group.

## Figures and Tables

**Table 1:** Background characteristics.

	LHGS Group (*n* = 21)	HHGS Group (*n* = 20)	*p*-Value
Age (years)	75.8 ± 7.1	77.4 ± 6.7	0.47
Male sex (*n*, %)	13, 62%	12, 60%	1
Grip strength (kg)	18.3 ± 4.6	26.5 ± 5.4	<0.001
Grip strength in males (kg)	20.9 ± 3.8	30.1 ± 3.0	<0.001
Grip strength in females (kg)	14.0 ± 1.5	21.0 ± 2.7	<0.001
MMSE score	24.3 ± 3.7 (*n* = 20)	26.5 ± 3.2 (*n* = 13)	0.04
Body mass index (kg/m*^2^*)	24.2 ± 3.5	25.5 ± 4.1	0.30
Waist circumference (cm)	90.4 ± 8.9	95.1 ± 12.5	0.18
Duration of DM (years)	18.7 ± 13.3	16.3 ± 11.25	0.52
Family history of DM (*n*, %)	12, 57%	13, 65%	0.75
Current smoking (*n*, %)	3, 14%	3, 15%	1
Habitual alcohol use (*n*, %)	1, 5%	3, 15%	0.34
Insulin use (*n*, %)	6, 29%	3, 15%	0.45
Statin use (*n*, %)	13, 62%	11, 55%	0.76
Hypertension (*n*, %)	15, 71%	15, 75%	1
Systolic BP (mmHg)	131.4 ± 15.4	133.2 ± 13.4	0.71
Diastolic BP (mmHg)	71.8 ± 8.7	68.4 ± 12.9	0.32
Dyslipidemia (*n*, %)	16, 76%	15, 75%	1
History of cardiovascular disease (*n*, %)	4, 19%	2, 10%	0.66
History of cerebrovascular disease (*n*, %)	2, 10%	2, 10%	1
History of peripheral artery disease (*n*, %)	4, 19%	2, 10%	0.66

Data represents mean ± standard deviation or number and percentage. BP, blood pressure; DM, diabetes mellitus; HHGS, higher hand grip strength; LHGS, lower hand grip strength; MMSE, mini-mental state examination.

**Table 2:** Diabetic microangiopathy and surrogate markers of atherosclerosis.

	LHGS Group (*n* = 21)	HHGS Group (*n* = 20)	*p*-Value
DPN (*n*, %)	16, 76%	8, 40%	0.03
CVR-R (%)	2.85 ± 2.75 (*n* = 18)	3.03 ± 2.00 (*n* = 18)	0.83
CVR-R < 2% (*n*, %)	10, 56% (*n* = 18)	5, 27% (*n* = 18)	0.18
Retinopathy (*n*, %)	10, 50% (*n* = 20)	6, 30%	0.27
Nephropathy			
Stage 1, pre-nephropathy (*n*, %) Urinary albumin < 30 mg/gcre or mg/day	4, 19%	8, 40%	0.18
Stage 2, incipient nephropathy (*n*, %) Urinary albumin 30–299 mg/gcre or mg/day	13, 62%	8, 40%	0.22
Stage 3, overt nephropathy (*n*, %) Urinary albumin ≥ 300 mg/gcre or mg/day Urinary protein ≥ 0.5 g/gcre or g/day	1, 5%	2, 10%	0.61
Stage 4, kidney failure (*n*, %) eGFR < 30 mL/min/1.73 m^2^	3, 14%	2, 10%	1
Stage 5, dialysis therapy (*n*, %)	0, 0%	0, 0%	1
Maximum IMT (mm)	2.45 ± 1.18	2.42 ± 0.84	0.92
Minimal ABI	1.01 ± 0.19	1.05 ± 0.16	0.54
Average ABI	1.04 ± 0.16	1.08 ± 0.15	0.49
Average CAVI	9.83 ± 1.43	9.82 ± 1.10	0.99

Data represent mean ± standard deviation or number and percentage. ABI, ankle-brachial index; CAVI, cardio-ankle vascular index; CVR-R, coefficient of variation of the R-R interval; DPN, diabetic peripheral neuropathy; eGFR, estimated glomerular filtration rate; HHGS, higher hand grip strength; IMT, intima-media thickness; LHGS, lower hand grip strength.

**Table 3:** Laboratory parameters.

	LHGS Group (*n* = 21)	HHGS Group (*n* = 20)	*p*-Value
HbA1c (%)	8.8 ± 1.5	8.9 ± 1.4	0.87
GA (%)	25.6 ± 5.7	26.3 ± 6.5	0.73
TLC (/µL)	1651 ± 544	1639 ± 580	0.95
Hemoglobin (g/dL)	12.5 ± 1.4	13.0 ± 1.3	0.21
Albumin (g/dL)	4.0 ± 0.5	4.1 ± 0.4	0.61
AST (U/L)	25.8 ± 18.2	25.3 ± 12.7	0.91
ALT (U/L)	22.2 ± 22.5	20.9 ± 12.9	0.82
UA (mg/dL)	5.0 ± 1.4	4.9 ± 1.3	0.84
Total cholesterol (mg/dL)	169.0 ± 43.2	174.6 ± 33.8	0.65
HDL cholesterol (mg/dL)	48.9 ± 12.7	51.1 ± 12.3	0.58
Directly measured LDL cholesterol (mg/dL)	92.8 ± 35.7	97.3 ± 30.4	0.66
Calculated LDL cholesterol (mg/dL)	94.5 ± 36.9	101.5 ± 33.7	0.53
Triglyceride (mg/dL)	127.9 ± 57.8	110.1 ± 44.7	0.28
Creatinine (mg/dL)	0.91 ± 0.49	0.96 ± 0.49	0.74
eGFR (mL/min/1.73 m^2^)	66.7 ± 23.1	62.3 ± 22.9	0.54
Sodium (mEq/L)	140.2 ± 4.6	141.4 ± 2.4	0.30
Potassium (mEq/L)	4.2 ± 0.6	4.4 ± 0.4	0.31
Chloride (mEq/L)	102.7 ± 4.7	105.0 ± 2.2	0.05
Corrected calcium (mg/dL)	9.1 ± 0.5	9.0 ± 0.4	0.49
Phosphorus (mg/dL)	3.8 ± 0.4	3.3 ± 0.5	<0.01

Data represent mean ± standard deviation. AST, aspartate aminotransferase; ALT, alanine aminotransferase; eGFR, estimated glomerular filtration rate; GA, glycated albumin; HbA1c, glycated hemoglobin; HDL, high-density lipoprotein; HHGS, higher hand grip strength; LDL, low-density lipoprotein; LHGS, lower hand grip strength; TLC, total lymphocyte count; UA, uric acid.

**Table 4:** Multiple linear regression analysis using grip strength as the dependent variable.

	*β*-Value	*p*-Value	*t*-Statistic	95% Confidence Interval	
				Lower	Upper
Phosphorus (mg/dL)	−7.10	<0.001	−3.82	−10.88	−3.33
eGFR (mL/min/1.73 m^2^)	−0.07	0.10	−1.69	−0.16	0.01
Age (years)	−0.22	0.16	−1.43	−0.52	0.09
BMI (kg/m^2^)	0.28	0.24	1.19	−0.20	0.79
HbA1c (%)	−0.12	0.85	−0.19	−1.42	1.17

Dependent variable is grip strength (kg). Independent variables are phosphorus, eGFR, age, body mass index, and HbA1c. Adjusted R-squared is 0.25. β, regression coefficient; BMI, body mass index; eGFR, estimated glomerular filtration rate; HbA1c, glycated hemoglobin.

## Supplementary Materials

The following are available online at https://www.mdpi.com/2308-3417/5/2/33/s1, Figure S1: Correlation analysis between mini-mental examination score and hand grip strength. The vertical axis indicates the mini-mental examination score. The horizontal axis indicates grip strength (kg). Pearson’s correlation coefficient of *r* = −0.226 (*p* = 0.21).

## References

[1] Cruz-Jentoft A.J., Bahat G., Bauer J., Boirie Y., Bruyere O., Cederholm T., Cooper C., Landi F., Rolland Y., Sayer A.A. (2019). Sarcopenia: Revised European consensus on definition and diagnosis. Age Ageing.

[2] Morley J.E., Vellas B., van Kan G.A., Anker S.D., Bauer J.M., Bernabei R., Cesari M., Chumlea W.C., Doehner W., Evans J. (2013). Frailty consensus: A call to action. J. Am. Med. Dir. Assoc..

[3] Umegaki H. (2016). Sarcopenia and frailty in older patients with diabetes mellitus. Geriatr. Gerontol. Int..

[4] Wong E., Backholer K., Gearon E., Harding J., Freak-Poli R., Stevenson C., Peeters A. (2013). Diabetes and risk of physical disability in adults: A systematic review and meta-analysis. Lancet Diabetes Endocrinol..

[5] Yanase T., Yanagita I., Muta K., Nawata H. (2018). Frailty in elderly diabetes patients. Endocr. J..

[6] Park S.W., Goodpaster B.H., Strotmeyer E.S., de Rekeneire N., Harris T.B., Schwartz A.V., Tylavsky F.A., Newman A.B. (2006). Decreased muscle strength and quality in older adults with type 2 diabetes: The health, aging, and body composition study. Diabetes.

[7] Wang C.P., Hazuda H.P. (2011). Better glycemic control is associated with maintenance of lower-extremity function over time in Mexican American and European American older adults with diabetes. Diabetes Care.

[8] Chen L.K., Liu L.K., Woo J., Assantachai P., Auyeung T.W., Bahyah K.S., Chou M.Y., Chen L.Y., Hsu P.S., Krairit O. (2014). Sarcopenia in Asia: Consensus report of the Asian Working Group for Sarcopenia. J. Am. Med. Dir. Assoc..

[9] Haneda M., Noda M., Origasa H., Noto H., Yabe D., Fujita Y., Goto A., Kondo T., Araki E. (2018). Japanese Clinical Practice Guideline for Diabetes 2016. J. Diabetes Investig..

[10] Haneda M., Utsunomiya K., Koya D., Babazono T., Moriya T., Makino H., Kimura K., Suzuki Y., Wada T., Ogawa S. (2015). A new Classification of Diabetic Nephropathy 2014: A report from Joint Committee on Diabetic Nephropathy. J. Diabetes Investig..

[11] Kanda Y. (2013). Investigation of the freely available easy-to-use software ‘EZR’ for medical statistics. Bone Marrow Transplant..

[12] Clouston S.A., Brewster P., Kuh D., Richards M., Cooper R., Hardy R., Rubin M.S., Hofer S.M. (2013). The dynamic relationship between physical function and cognition in longitudinal aging cohorts. Epidemiol. Rev..

[13] Chou M.Y., Nishita Y., Nakagawa T., Tange C., Tomida M., Shimokata H., Otsuka R., Chen L.K., Arai H. (2019). Role of gait speed and grip strength in predicting 10-year cognitive decline among community-dwelling older people. BMC Geriatr..

[14] Stenholm S., Sainio P., Rantanen T., Koskinen S., Jula A., Heliovaara M., Aromaa A. (2007). High body mass index and physical impairments as predictors of walking limitation 22 years later in adult Finns. J. Gerontol. Ser. A Biol. Sci. Med. Sci..

[15] Kim H., Suzuki T., Kim M., Kojima N., Yoshida Y., Hirano H., Saito K., Iwasa H., Shimada H., Hosoi E. (2015). Incidence and predictors of sarcopenia onset in community-dwelling elderly Japanese women: 4-year follow-up study. J. Am. Med. Dir. Assoc..

[16] Hubbard R.E., Lang I.A., Llewellyn D.J., Rockwood K. (2010). Frailty, body mass index, and abdominal obesity in older people. J. Gerontol. Ser. A Biol. Sci. Med. Sci..

[17] Fukuoka Y., Narita T., Fujita H., Morii T., Sato T., Sassa M.H., Yamada Y. (2019). Importance of physical evaluation using skeletal muscle mass index and body fat percentage to prevent sarcopenia in elderly Japanese diabetes patients. J. Diabetes Investig..

[18] Andersen H., Nielsen S., Mogensen C.E., Jakobsen J. (2004). Muscle strength in type 2 diabetes. Diabetes.

[19] Almurdhi M.M., Reeves N.D., Bowling F.L., Boulton A.J., Jeziorska M., Malik R.A. (2016). Reduced Lower-Limb Muscle Strength and Volume in Patients with Type 2 Diabetes in Relation to Neuropathy, Intramuscular Fat, and Vitamin D Levels. Diabetes Care.

[20] Gundmi S., Maiya A.G., Bhat A.K., Ravishankar N., Hande M.H., Rajagopal K.V. (2018). Hand dysfunction in type 2 diabetes mellitus: Systematic review with meta-analysis. Ann. Phys. Rehabil. Med..

[21] Lima K.C.A., Borges L.D.S., Hatanaka E., Rolim L.C., de Freitas P.B. (2017). Grip force control and hand dexterity are impaired in individuals with diabetic peripheral neuropathy. Neurosci. Lett..

[22] Moon H., Chin H.J., Na K.Y., Joo K.W., Kim Y.S., Kim S., Han S.S. (2019). Hyperphosphatemia and risks of acute kidney injury, end-stage renal disease, and mortality in hospitalized patients. BMC Nephrol..

[23] Allen D.G., Westerblad H. (2001). Role of phosphate and calcium stores in muscle fatigue. J. Physiol..

[24] Dahlstedt A.J., Katz A., Westerblad H. (2001). Role of myoplasmic phosphate in contractile function of skeletal muscle: Studies on creatine kinase-deficient mice. J. Physiol..

[25] Chen Y.Y., Kao T.W., Chou C.W., Wu C.J., Yang H.F., Lai C.H., Wu L.W., Chen W.L. (2018). Exploring the Link between Serum Phosphate Levels and Low Muscle Strength, Dynapenia, and Sarcopenia. Sci. Rep..

[26] Lederer E. (2014). Regulation of serum phosphate. J. Physiol..

[27] Kuro-o M. (2010). A potential link between phosphate and aging—Lessons from Klotho-deficient mice. Mech. Ageing Dev..

[28] Beben T., Ix J.H., Shlipak M.G., Sarnak M.J., Fried L.F., Hoofnagle A.N., Chonchol M., Kestenbaum B.R., de Boer I.H., Rifkin D.E. (2016). Fibroblast Growth Factor-23 and Frailty in Elderly Community-Dwelling Individuals: The Cardiovascular Health Study. J. Am. Geriatr. Soc..

[29] Visser M., Deeg D.J., Lips P. (2003). Low vitamin D and high parathyroid hormone levels as determinants of loss of muscle strength and muscle mass (sarcopenia): The Longitudinal Aging Study Amsterdam. J. Clin. Endocrinol. Metab..

