# Supplementary material for: Inverse Correlation Between Grip Strength and Serum Phosphorus: A Retrospective Observational Study in Japanese Elderly with Poorly Controlled Type 2 Diabetes
Source: Geriatrics (Basel). 2020 May 19;5(2):33. doi: 10.3390/geriatrics5020033 (PMC7346010; doi:10.3390/geriatrics5020033)
Supplement: Supplementary file 1 [file geriatrics-05-00033-s001.pdf]

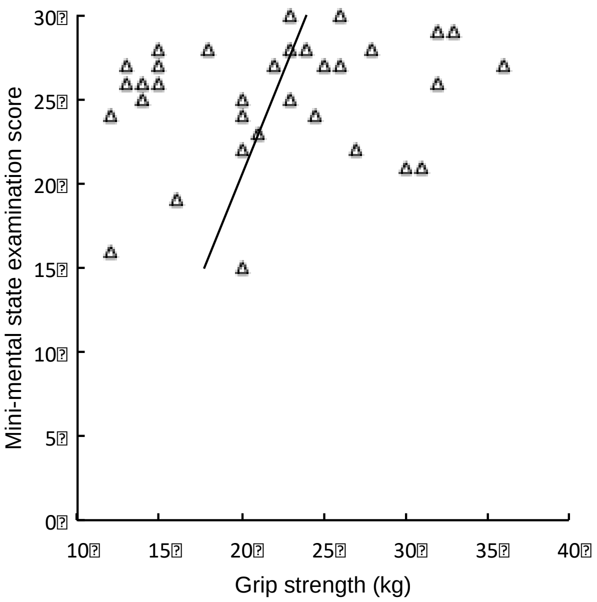

Figure S1. Correlation analysis between mini-mental examination score and hand grip strength. The vertical axis indicates the mini-mental examination score. The horizontal axis indicates grip strength (kg). Pearson's correlation coefficient of $r = -0.226$ ($p = 0.21$).
